# Assessment of Takayasu arteritis in routine practice with PETVAS, an 18F-FDG PET quantitative scoring tool

**DOI:** 10.3906/sag-2106-129

**Published:** 2021-09-30

**Authors:** Sema KAYMAZ-TAHRA, Salih ÖZGÜVEN, Ali Uğur ÜNAL, Fatma ALİBAZ-ÖNER, Tunç ÖNEŞ, Tanju Yusuf ERDİL, Haner DİRESKENELİ

**Affiliations:** 1 Division of Rheumatology, Department of Internal Medicine, School of Medicine, Marmara University, İstanbul, Turkey; 2 Department of Nuclear Medicine, School of Medicine, Marmara University, İstanbul, Turkey

**Keywords:** Large-vessel vasculitis, imaging, positron emission tomography, fluorodeoxyglucose, Takayasu arteritis

## Abstract

**Background/aim:**

The aim of this study was to evaluate the value of the PET vascular activity score (PETVAS) during the follow-up of patients with Takayasu arteritis.

**Materials and methods:**

Takayasu arteritis patients who underwent 18F-Fluorodeoxyglucose (FDG) PET imaging were evaluated retrospectively. In 8 patients both 1 and 2-h imagings were also performed prospectively. For PETVAS, 9 arterial areas were scored between 0–3 according to the FDG uptake.

**Results:**

Forty-six images of 34 patients were evaluated. PETVAS was higher in patients with clinically active disease (p = 0.03) and in the C-reactive protein (CRP) elevated group among clinically inactive patients (p = 0.0015). PETVAS correlated with CRP (p = 0.003, r = 0.53) and erythrocyte sedimentation rate (p = 0.005, r = 0.41), whereas age, disease duration, immunosuppressive, and glucocorticoid (GC) treatments were not associated with PETVAS. First vs. 2nd-h PETVAS was similar in patients who had both 1st and 2nd h PET scans (p = 0.67).

**Conclusion:**

We observed higher PETVAS in patients with active disease and elevated acute phase reactants. Although scores in our study (performed at one-h) were lower compared to the original PETVAS study performed at two h, PETVAS seems to be a reliable tool to quantify FDG PET scores in routine practice.

## 1. Introduction

Takayasu arteritis (TAK) is a primary large-vessel vasculitis affecting aorta and its main branches [1]. Conventional digital subtraction angiography (DSA) which provides information about luminal changes used to be the gold standard imaging method for the diagnosis of TAK. However, as an invasive method which cannot delineate information about vessel-wall inflammation, DSA is mostly replaced by magnetic resonance angiography (MRA), computed tomography angiography (CTA), Doppler ultrasound (US) or positron emission tomography (PET) in recent years [2].

FDG PET/CT is a noninvasive, verified imaging modality to detect the regional distribution of 18F-FDG in oncological diseases and also has a promising value for large-vessel vasculitis (LVV). Interpretation of FDG uptake is based on the principle of determining metabolically active cells such as immune/inflammatory cells invading the vessel wall in LVV [3]. It can reflect the localization and degree of inflammation [4]. Previous studies have shown that FDG PET/CT is useful in the diagnosis of LVV with a specificity of up to 90% [5]. FDG PET/CT also has a role in detecting disease activity with a sensitivity of 70%–87% and specificity of 73%–87% [6–7]. Therefore, FDG PET/CT is recommended as one of the major imaging modalities for the diagnosis and monitoring of primary LVVs, such as Takayasu arteritis (TAK) and giant-cell arteritis.

However, interpretation of FDG PET/CT has some challenges. The main controversies in LVV are the lack of standardization in the definition of vascular inflammation and the time interval between the FDG administration and acquisition [8]. In recent ‘EULAR recommendations for the use of imaging in LVV in clinical practice’ a minimum of 60 min between intravenous FDG administration and acquisition is advised, but a delayed acquisition may be chosen to increase the sensitivity of detecting FDG uptake in LVV [2]. However, most studies with FDG PET/CT in the literature is performed at one-h and the data from studies comparing the first h and delayed acquisition is controversial [8–11].

Recently PETVAS (PET vascular activity score), a quantitative score assessed at 2-h of FDG uptake, is introduced for a standardized assessment of FDG PET/CT in LVV [12]. In this study, we aimed to evaluate the value of PETVAS (performed at one-h) during the initial diagnosis and follow-up of patients with TAK in routine practice.

## 2. Materials and methods

### 2.1. Patients

In this single-center study 34 patients who were diagnosed with Takayasu arteritis and underwent FDG PET/CT imaging during their routine follow-up were evaluated retrospectively. All patients fulfilled the American College of Rheumatology (ACR) 1990 classification criteria for Takayasu arteritis [13]. Demographic and clinical characteristics of the patients were recorded from patients’ charts. The study was approved by the local ethics committee (no: 09.2019.605). All patients provided written informed consent. The study was performed in accordance with the Declaration of Helsinki.

Physician’s Global Assessment (PGA) was used to determine clinical activity according to Kerr’s (NIH) criteria. Patients were accepted as active according to the Kerr’s criteria if two of the following four items were positive: systemic features with no other cause, elevated CRP, features of vascular ischemia or inflammation or typical angiographic features [14]. The IS and/or GC use and the doses of the treatments were recorded. GC doses were given in prednisolone equivalent doses.

### 2.2 FDG-PET/CT imaging technique

All patients had fasted for at least 6 h before FDG PET/CT imaging was performed and only oral hydration with glucose-free water was allowed. FDG PET/CT scans were performed consistent with European League Against Rheumatism (EULAR) and the European Association of Nuclear Medicine (EANM), the Cardiovascular Council of the Society of Nuclear Medicine and Molecular Imaging (SNMMI), and the PET Interest Group (PIG), and endorsed by American Society of Nuclear Cardiology recommendations (ASNC) [2,8]. The blood glucose level of the patients that were checked before the FDG administration is required to be below 126 mg/dL. All patients rested in a quiet and temperature-balanced room (20–22 °C) for 60 min after intravenous injection of 3.7 MBq (0.1 mCi)/kg of FDG. Only oral contrast, not an intravenous agent, was administered according to our local departmental protocols. Image acquisition was performed using an integrated PET/CT system (Discovery-16LS, GE Healthcare). PET images were obtained from mid-skull to below the knees in 3D mode and reconstructed in transverse, coronal and sagittal planes. For attenuation correction and anatomic orientation, a low-dose CT scan was performed. After the image acquisition, data were transferred to a workstation (Advantage Windows Workstation 4.5, GE Healthcare) for processing and interpretation.

Image acquisition was performed one h after FDG administration. In 8 patients both 1 and 2-h images were acquired prospectively to compare the characteristics of images in early and delayed time intervals.

### 2.3. Image interpretation

**Figure 1:**
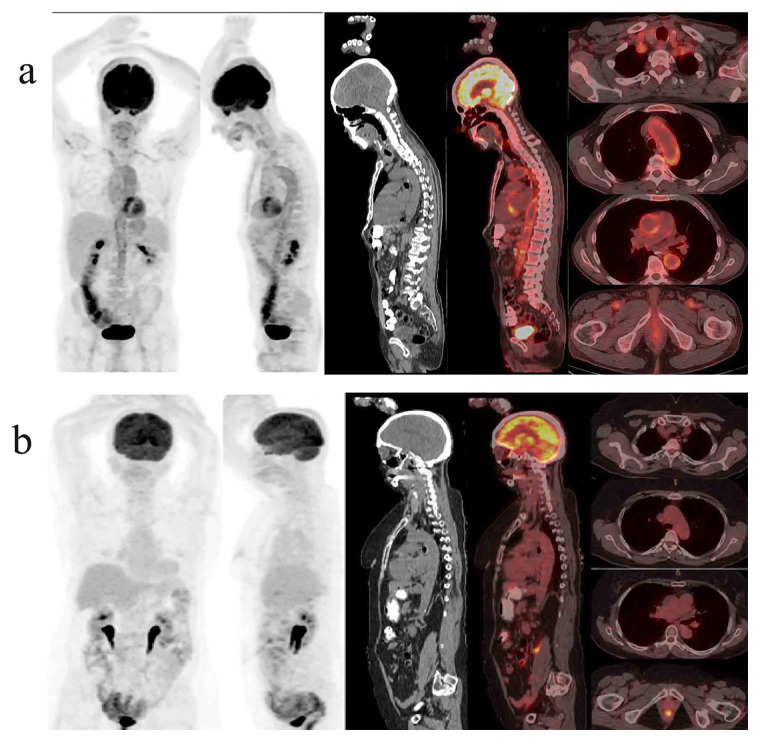
Representative PET/CT images showing maximum (PETVAS = 27) (a) and minimum (PETVAS = 0) (b) scores.

Two independent nuclear physicians who were blinded to the clinical data, interpreted the images. A total of 15 arterial areas (ascending aorta, aortic arch, descending thoracic aorta, abdominal aorta, innominate, carotid arteries, subclavian arteries, axillary arteries, iliac arteries, and femoral arteries) were evaluated. In the PETVAS scoring, performed at one h of the FDG uptake, 9 arterial areas (ascending aorta, aortic arch, descending thoracic aorta, abdominal aorta, right carotid artery, left carotid artery, innominate artery, right subclavian artery and left subclavian artery) were scored between 0 and 3 according to the FDG uptake separately, as originally suggested (scale 0–27) [12]. The FDG uptake is graded as grade 0 = no uptake, grade 1 = less than liver involvement, grade 2 = equal involvement to the liver, grade 3 = greater than liver involvement (representative PET/CT images showing maximum and minimum PETVAS are presented in Figures 1a and 1b).

The ‘visual analysis’ (VA) using the liver FDG uptake as the reference was also assessed and compared with PETVAS. If the uptake of an arterial area is equal to or greater than liver uptake (≥ grade 2) that arterial area is considered as ‘active’ or ’positive’. ‘Active PET-CT’ is defined as the presence of at least one active arterial area.

### 2.4. Statistical analysis

For continuous normally-distributed variables, results are expressed as mean ± SD. In the case of nonnormally distribution median (min-max) values are presented. Chi-square test and Fischer’s exact test were used to compare the categorical data. Independent continuous variables were compared with the Mann-Whitney U test. Wilcoxon signed rank test was used to compare the first and second-h PETVAS of the prospective group. A p value of <0.05 was considered statistically significant. Correlation analyses were performed with Spearman’s rank method. Logistic regression analysis was used to assess variables associated with positive PET results. Logistic regression analysis was performed for two different dependent variables. In the first analysis, active PET according to visual analysis (VA) was used as the dependent variable. Female gender, age, glucocorticoid dose, disease duration, BMI, active disease, immunosuppressive use, ESR and CRP were evaluated as covariates. In the second logistic regression analysis PETVAS 5.5 which had 76% specificity and 70.6% sensitivity for active disease was used as a threshold. The predictive factors for PETVAS ≥ 5.5 were evaluated. Statistical package for the social sciences 20.0 (SPSS, Chicago, IL, USA) was used to perform the analysis.

## 3. Results

**Figure 2:**
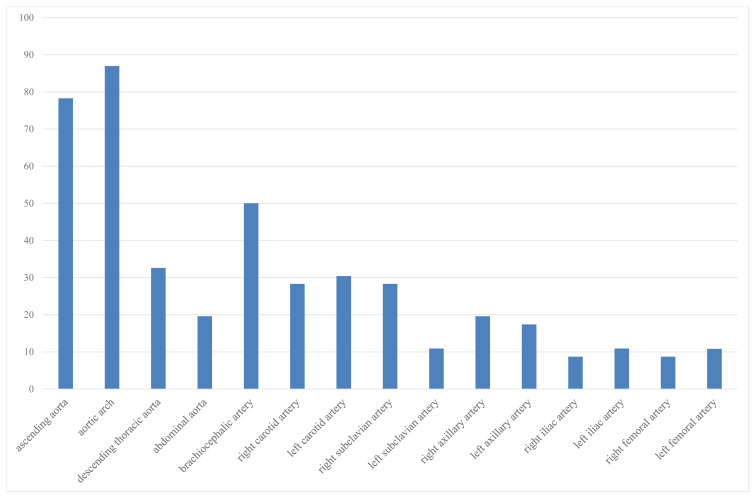
Distribution of vascular FDG uptake in Takayasu arteritis patients.

Fourty-six imagings of 34 patients (F/M: 28/6) were evaluated. Mean disease duration was 9.3 ± 6.5 years and the mean age was 40.5 ± 15.1 years. Median CRP level was 16.3 (2–126) mg/L. Patients characteristics at the time of imaging are given in Table 1. In the majority of patients, aortic arch (87%) was involved followed by ascending aorta (78%) and brachiocephalic artery (50%) (Figure 2). Median 3.5 (0–15) arterial regions were involved.

**Figure 3:**
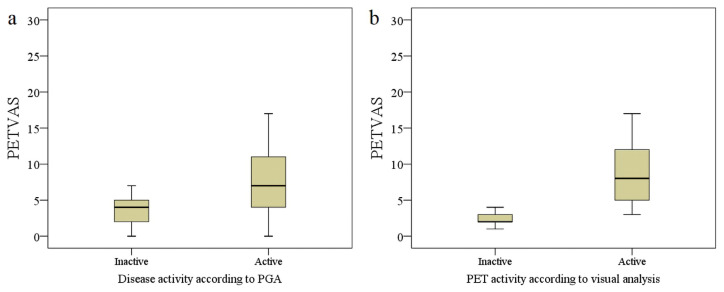
PETVAS values of patients according to clinical disease activity and PET positivity determined with visual analysis. PETVAS was significantly higher in patients who were clinically active (p = 0.03) (a) and who had active PET according to visual analysis (p < 0.01) (b).

Twenty-five (54%) imagings were evaluated to be PET-positive according to visual analysis and median PETVAS was 4.5 (0–27). Patients who had a positive PET assessed with visual analysis (VA) had higher PETVAS than patients who were VA inactive (median score 8.0 vs. 2.0, p < 0.001) (Figures 3a and 3b). Twelve (70%) clinically active patients had positive PET. Interestingly PET was also positive in 45% (13/29) of the patients who had inactive disease. Active PET-CT according to visual analysis had a sensitivity of 70.6% and specificity of 55.2% in the distinction of clinically active patients from inactive patients. PETVAS was also significantly higher in patients who were considered to have active disease (median PETVAS 7 (0–27) vs. 4 (0–17), p = 0.03). When we used a cut-off value of 5.5 for PETVAS the specificity was 75.9% (area under curve = 0.69).

PETVAS of the patients who experienced at least one relapse after imaging and who remained in remission were similar in the whole group (median score 5 (0–27) vs. 4 (0–26), p = 0.11). PETVAS was also similar between relapsing and nonrelapsing patients (PETVAS 4 (0–17) vs. 3 (0–4), p = 0.12) among the clinically inactive group. No difference in PETVAS was present between patients who were taking GC vs. non-GC use (median PETVAS: GC+: 4 (0–17) vs. GC−: 5 (1–27), p = 0.90) or according to immunosuppressive (ISs) use (ISs+: 4 (0–17) vs. ISs−: 5 (2–27), p = 0.55). PETVAS also was not correlated with age (p = 0.66, r = 0.07) and disease duration (p = 0.67, r = −0.06).

Eleven of 13 (85%) inactive patients with VA PET positivity were in the elevated CRP subgroup. Median PETVAS values were also significantly higher in the CRP elevated inactive group (p = 0.0015) (Table 2). A positive correlation was observed between PETVAS and both CRP (p = 0.003, r = 0.53) and ESR (p = 0.005, r = 0.41) levels.

In univariable logistic regression analysis, only CRP (OR (95% CI): 1.05 (1.01–1.11), p = 0.02) was associated with active PET according to visual analysis, but ESR (OR (95% CI): 1.01 (0.99–1.04), p = 0.10) and active disease (OR (95% CI): 2.95 (0.82–10.5), p = 0.09) had a trend towards an association. In multivariable analysis, none of the variables showed an association with only CRP having a trend for association (OR (95% CI): 1.05 (0.99–1.11), p = 0.06). When we evaluated variables associated with higher PETVAS with a threshold of 5.5, although ESR and CRP levels were associated with PETVAS > 5.5 in univariable analyses, only CRP was found to be a predictive factor for higher PETVAS values in multivariable analysis (Table 3).

In 43 (94%) imagings PETVAS was >0 and the treatment was changed in 25 (58%) patients. Among patients with a PETVAS >0, nine patients were clinically inactive but had high CRP levels. FDG PET/CT was performed during diagnostic period for 5 patients. In 20 (49%) of the remaining 41 images, PET-CT was positive and treatment was changed in 18 (90%) of the PET-positive patients. Nine out of 11 (82%) clinically inactive patients with elevated CRP and positive PET underwent a treatment change which was defined as GC dose increase and/or ISs change.

Twelve patients had follow-up PET scans with a mean 29.5 ± 14.5 months’ interval between baseline and follow-up imaging. At baseline imaging 10 of 12 (83%) patients with follow-up imaging had positive PET/CT according to VA. Among this group, the median GC dose was 5 mg (0–7.5) in VA active PET/CT group and 1.25 mg (0–2.5) in VA inactive group. Immunosuppressive (IS) treatment was increased in 8 (8/10, 80%) patients with active PET at baseline. Among these 8 patients with treatment change, treatment was switched to another csDMARD in 4 patients with a conventional disease-modifying antirheumatic drug (csDMARD) therapy. IS treatment and GC therapy were added to 2 patients who were not on IS treatment during the first imaging whereas treatment was increased to TNF inhibitors for 2 patients with active PET at initial imaging. Median PETVAS at follow-up visit significantly decreased after treatment change compared to baseline imaging (PETVAS 1st vs. 2nd imaging: 9 (5–17) vs. 5.5 (2–17), p = .0.04). PETVAS was similar in baseline and follow-up images in 4 patients without treatment change (Median PETVAS 1st vs. PETVAS 2nd 3.5(2–5) vs. 2.5 (2–14), p = 0.52).

**Figure 4:**
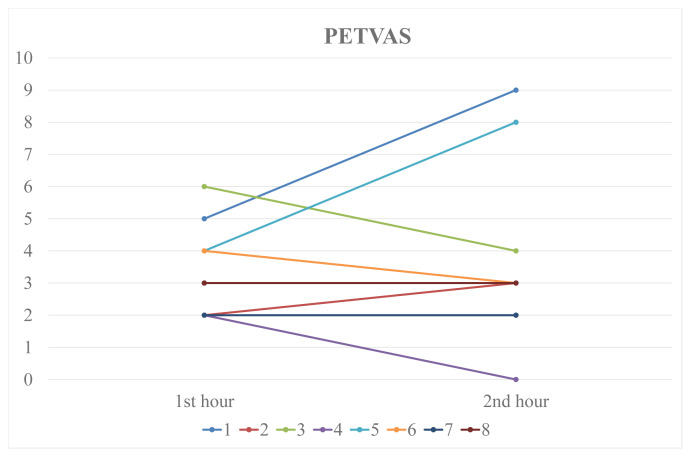
Comparison of 1^st^ and 2^nd^-h PETVAS.

In 8 patients PET-CT was performed both in the first and second h of the FDG uptake. Median PETVAS was similar in 1st and 2nd h (3.5 (2–6) vs. 3.0 (0–9), p = 0.67). In the 2nd h scans, PETVAS increased in 3, decreased in 3 and was stable in 2 patients compared to the 1st-h uptake (Figure 4). According to VA one imaging was considered active in 1st h and 2 imagings were active in the 2nd h of FDG uptake (p = 0.25). In 28% of the imagings which had a PETVAS > 0 (12/43), a PET involvement of an artery other than the arteries used for assessing PETVAS was observed.

## 4. Discussion

In our study, FDG PET/CT assessment with PETVAS (assessed at one h) demonstrated higher scores in patients with Takayasu arteritis who were considered clinically active or had increased CRP levels. However, the scores were lower compared to the original PETVAS performed at two h and the uptakes in the 1st and 2nd h after FDG administration were not different in our study.

Disease activity assessment is challenging in TAK. Imaging modalities are not routinely recommended for patients in clinical remission in recent EULAR guidelines [2]. However, if the clinical and laboratory findings are not adequately conclusive, imaging methods can be required before the final clinical judgment. FDG PET/CT is a noninvasive, functional imaging method that has promising results in the assessment of TAK and is recommended in the diagnosis and follow-up of TAK patients [2]. In our routine clinical practice, we choose to use FDG PET/CT imaging mostly for patients who have persistent APR response without clear clinical signs and symptoms of ischemia or patients with typical clinical findings in the early disease phase, mostly in the diagnostic period.

There are no precise criteria for the assessment of FDG uptake for PET-CT in LVV. However, a number of FDG PET/CT interpretation methods have been developed. Visual analysis is a commonly used semiquantitative method, comparing the interested arterial area uptake with the background uptake of liver. An uptake equal to or higher than liver uptake corresponds to active vascular involvement [15–19]. The highest standard uptake value (SUV) of the arterial territories (SUVmax), the ratio of SUVmax to SUV liver (SUVratio) and mean SUV of the arterial areas (SUVmean) are also used for quantitative analysis of the FDG uptake [20–21]. Finally, PETVAS, which is a total quantitative score of the most commonly involved 9 arteries in LVV, is developed recently to increase the discriminative value of PET assessment and standardize clinical and therapeutic studies [12].

In the study in which PETVAS was originally described, Grayson et al. observed that active FDG PET/CT differentiated clinically active LVV patients from comparators with a sensitivity and specificity of over 80%. However, more than half of the patients (58%) who were in remission clinically according to NIH criteria were also interpreted to have active FDG PET/CT and the specificity of FDG PET/CT in distinguishing clinically active patients from inactives was only 42%. In the comparator group who did not have an LVV diagnosis, 17% of patients were found to have active vasculitic lesions. When PETVAS was used with a cut-off value of >20 the sensitivity increased to 68% and specificity to 71% [12]. PETVAS was also used in a study in which the relationship between different disease activity measures, such as patient reported outcomes, physician reported outcomes, laboratory outcomes and imaging outcomes in LVV was assessed. PETVAS, which was considered as the imaging outcome, had the strongest association in distinguishing the active disease defined by PGA [22].

In another study, Zhang et al. showed that SUV max, SUV mean and SUV ratios were significantly higher in clinically active group compared to the inactives and with a 2.1 SUV max cut-off they reported 86.2% sensitivity and 90% specificity [21]. Schramm et al. also described 68.4% sensitivity and 91.3% specificity according to VA for active disease [23]. In contrast to these studies, Arnoud et al. reported no correlation between clinical activity and FDG uptake [17]. Kang et all compared the performance of PETVAS and SUV max for disease activity assessment in TAK patients. They found that PETVAS, which provides a global PET activity assessment, was superior for distinguishing active disease compared to regional assessment with SUV max [24]. In the current study, we also observed that PETVAS was significantly higher in clinically active patients. A positive FDG PET/CT according to VA is observed in clinically active patients with a sensitivity of 70.6% and specificity of 55.2%. With a PETVAS threshold score of 5.5, the specificity increased to 76% with a sensitivity of 70.6%.

Although most of the studies in the literature suggest that clinical activity is associated with FDG PET/CT positivity, the implications of an active FDG PET/CT assessment in clinically inactive patients are not well described. It may be a sign of ongoing subclinical inflammation which was previously shown in some surgical specimens [14, 25]. Lee et al. reported that in clinically inactive patients with an active vasculitic FDG uptake at baseline, follow-up scans showed a decrease in FDG uptake with less vasculitic extension after immunosuppressive treatments [15]. Grayson et al. found active PET in nearly 60% of patients who were clinically inactive. The most likely cause was suggested as subclinical vasculitis rather than vascular remodelling and atherosclerosis [12]. Similar to these results 45% of our clinically inactive patients had active FDG PET/CT and most of these patients had elevated CRP levels (85%) despite lack of clinical findings.

CRP is a commonly used biomarker for disease activity assessment in TAK. However, its association with FDG PET/CT findings is variable in the literature. In a recent systematic review, when CRP effect on PET positivity was evaluated [26], five out of nine studies [16, 18–19, 27–28] reported an association between CRP and FDG PET/CT while other four studies [15, 17, 20, 29] found no association. Therefore, the authors concluded the effect of CRP on FDG PET/CT positivity only as ‘moderate’. We also observed a correlation between CRP and visual analysis in clinically inactive patients which led to treatment changes in our patient group. When a PETVAS of >5.5 was used as a cut-off, logistic regression analysis also demonstrated a weak but significant association with CRP. In 82% of the clinically inactive patients with elevated CRP and positive PET, treatment was changed in our patients. Therefore, isolated increased acute phase response with active PET findings seems to have an impact on treatment decisions. In such patients in whom the clinical and laboratory findings are not sufficient, the recommended imaging methods (MR angiography (MRA), CT angiography (CTA), and PET CT) could be chosen to determine the clinical activity. MRA can detect luminal changes and prestenotic lesions such as contrast enhancement, vessel wall thickening and periarterial lesions without radiation exposure [2]. CTA is useful in the assessment of active disease by demonstrating wall thickening and low attenuation ring which is assessed in the late contrast phase and has a high specificity for active disease [30]. PET CT is also a good option for detecting subclinical inflammation and may identify the focus of vascular inflammation earlier which may be an advantage for PET over other imaging modalities [2]. In summary, these patients should be followed closely and when needed the most suitable and available imaging method should be chosen by the clinician in individual basis.

PETVAS has also been assessed in terms of being a predictor of clinical relapses. Grayson et al. reported that higher PETVAS was associated with higher relapse rates during follow-up in LVV patients [12]. In a retrospective study including 17 LVV patients, the intensity of FDG uptake in baseline FDG PET/CT and extension of active vasculitic involvement were significantly higher in patients with the relapsing disease [31]. In contrast, Blockmans et al. observed no association of relapses with FDG PET/CT activity in biopsy-proven giant cell arteritis (GCA) patients [32]. Consistent with these results, PETVAS was similar between patients who had a future relapse vs. nonrelapsing disease in our study.

Data from previous studies suggest that GC treatment reduces the FDG uptake [12, 32–34] and the uptake decreases with the duration of GC treatment [33, 35]. However, GC use also causes an increase in liver FDG uptake which may cause underestimation of vascular uptake [36]. Less is known about the effect of immunosuppressives on vascular FDG PET/CT assessments, but some studies reported improvement in FDG uptake with glucocorticoid-sparing agents [34, 37–38]. In a recent study, a decrease in PETVAS was observed after GC and/or ISs treatments [34]. However, there was no difference in PETVAS between patients according to GC or IS use in our study, which might be explained by our low median GC doses.

Standardization of optimal timing in FDG PET/CT in LVV is still lacking. Delayed acquisitions of FDG PET/CT imagings may increase the sensitivity of detecting vasculitis as a result of an increase in vessel-wall uptake and a decrease in the blood pool uptake by longer timing [9, 12]. In a prospective study with 43 patients who were being evaluated with the suspicion of LVV, FDG PET/CT was performed 180 min after FDG injection and semiquantitative SUV values and target to background ratio (TBR) were better in differentiating LVV patients from the controls [11]. However, in another study evaluating PET activity in both 60 min and 180 min of FDG uptake, although SUV max, SUV mean and TBR increased with time, after correction for partial volume effects with contrast-enhanced CT (CECT) assistance, the SUV values and TBR were similar in both time intervals [10]. Quinn et al. evaluated one-h time-point PET-MR and two-h time-point PET-CT scans of 69 LVV patients. The percentage of positive PET scans increased from 56% to 77% and clinically active disease, which was defined as presence of clinical features directly assigned to vasculitis, was more common in the only delayed-time-point PET-positive patients. As there was a strong correlation in SUV sum values between one and two-h uptake, they reported that the same arterial territories were involved with greater SUV values at two-h time point [39].

PET studies in LVV patients were mostly performed at 60 min of acquisition in the literature. Our results showed no difference in 1st and 2nd-h images. In 1st h evaluation, one out of eight FDG PET/CT scans was active according to VA. Two patients had active FDG PET/CT according to VA in 2nd-h evaluation which was not statistically significant compared to the 1st h. Therefore, there is not strong enough evidence about delayed PET-CT imaging for detecting vasculitis in our study. Delayed imaging requires prolonged waiting times, which makes PET-CT imaging less convenient in routine clinical practice.

PETVAS values in our study were lower than reported by Grayson et al. Also, Kang et al., who reported a mean PETVAS value of 9.2, found lower numerical PETVAS values than National Institutes of Health (NIH) study [24]. Different patient characteristics may be a possible explanation for these variable results. Our TAK patient group who were mostly under ISs treatment for a long time had a younger mean age and a longer disease duration with a similar percentage of glucocorticoid use compared to NIH study cohort. Some technical reasons such as PET-CT machine properties and variability between readers among institutions may also explain lower PETVAS values in our study. We, therefore, think that a standardization study of FDG PET/CT quantitative assessment among nuclear medicine specialists is necessary before any quantitative PET/CT tool is widely accepted.

The main strength of our study was that we have reported our real-life experience in our TAK patients without any interventions in contrast to clinical trials. However, our study had some limitations. Retrospective design and use of PETVAS in routine practice limit our conclusions. A control group to detect sensitivity and specificity of the one-h PETVAS distinguishing TAK patients from comparators is also lacking. Finally, all patients did not have a concomitant angio-CT or MRA imaging showing vascular luminal changes at the time of disease activity assessment which is included in NIH criteria. This may have resulted in underestimating the number of patients with active disease.

In conclusion, the current study reflects the application of PETVAS to routine daily practice as a quantitative FDG PET/CT imaging tool in Takayasu arteritis. We observed higher PETVAS in patients with active clinical features or with elevated CRP levels in remission. Although our scores were lower compared to the original study, PETVAS seems a promising tool for the quantitative analysis of PET scans. Further studies are required with PETVAS, especially in therapeutic trials, to demonstrate its role better for the management of LVV.

## Figures and Tables

**Table 1:** The demographic and clinical features of Takayasu arteritis patients during the time of imaging.

Sex, (female/male)	28/6
Age, years, mean ± SD	40.5 ± 15.1
Disease duration, years, mean ± SD	9.3 ± 6.5
Cardiovascular risk factors	
* Diabetes, n (%)*	2 (6)
* Hypertension, n (%)*	15 (46)
* Hyperlipidemia, n (%)*	9 (27)
* Smoking-ever, n (%)*	11 (33)
* BMI, kg/m* *^2^* *, mean ± SD*	23.0 ± 3.6
Immunosuppressives n (%)	35 (76)
* Azathioprine*	18 (39)
* Methotrexate*	6 (13)
* Leflunomide*	9 (20)
* TNF-α inhibitors*	4 (9)
Glucocorticoid use present, n (%)	29 (63)
Glucocorticoid dose, mg†, median (range)	5 (2.5–80)
Acute phase reactants	
* ESR, mm/h*, mean ± SD	49.1 ± 28.0
* CRP, mg/L, median (range)*	16.3 (2–126)

† Prednisolone equivalent dose

**Table 2:** The disease activity and PET-CT interpretation according to visual analysis.

		Disease activity according to NIH criteria		p
		Active (n = 17)	Inactive (n = 29)	
Median PETVAS		7(0–27)	4(0–17)	0.03
Visual analyse	Active PET, n (%)	12 (48)	13 (52)	0.09
	Inactive PET, n (%)	5 (24)	16 (76)	
Inactive group (According to NIH criteria)				
		Elevated CRP (n = 16)	Negative CRP (n = 13)	p
Median PETVAS		5(2–17)	3(0–5)	0.0015
Visual analysis	Active PET, n (%)	11 (85)	2 (15)	0.008
	Inactive PET, n (%)	5 (31)	11 (69)	

**Table 3:** Variables associated with PETVAS> 5.5 scores in logistic regression analysis.

	Univariable analysis		Multivariable analysis†	
	OR (95% CI)	p	OR (95% CI)	p
Female sex	0.28 (0.04–1.79)	0.20	-	
Age, years	1.01 (0.96–1.06)	0.63	-	
Glucocorticoid dose, mg/d	0.97 (0.90–1.04)	0.40	-	
Disease duration, months	0.99 (0.98–1.00)	0.29	-	
BMI, kg/m2	0.96 (0.78–1.18)	0.72	-	
ISs present	0.84 (0.21–3.30)	0.80	-	
ESR, mm/h	1.02 (1.01–1.04)	0.04	0.99 (0.95–1.02)	0.61
CRP, mg/L	1.08 (1.02–1.14)	0.007	1.07 (1.01–1.14)	0.02

† Only variables with a p-value of <0.2 in univariable analysis were involved in multivariable analysis.

ESR: Erythrocyte sedimentation rate, CRP: C reactive protein, OR: Odds ratio, CI: Confidence interval.
